# Long-Term Surveillance of *Chlamydia psittaci* and West Nile Virus in Wild Birds from Central Spain (2013–2022)

**DOI:** 10.3390/microorganisms14010048

**Published:** 2025-12-25

**Authors:** Tania Ayllón, Irene Martínez, Gustavo Ortiz-Díez, Alejandro Navarro, Fernando Fuster, Andrés Iriso, Silvia Villaverde, José Lara, Nerea García

**Affiliations:** 1 VISAVET Health Surveillance Centre, Complutense University of Madrid, Av. Puerta de Hierro s/n, 28040 Madrid, Spain; irmartin@visavet.ucm.es (I.M.); angomez@ucm.es (A.N.); ngarciab@ucm.es (N.G.); 2 Department of Animal Health, Faculty of Veterinary Medicine, Complutense University of Madrid, Av. Puerta de Hierro s/n, 28040 Madrid, Spain; 3 Department of Animal Medicine and Surgery, Faculty of Veterinary Medicine, Complutense University of Madrid, Avda. Puerta de Hierro s/n, 28040 Madrid, Spain; gusortiz@ucm.es (G.O.-D.); jose.lara.zabia@madrid.org (J.L.); 4 Subdirección General de Seguridad Alimentaria y Sanidad Ambiental, Dirección General de Salud Pública, Consejería de Sanidad, Ronda de Segovia 52, 28005 Madrid, Spain; fernando.fuster@salud.madrid.org (F.F.); andres.iriso@salud.madrid.org (A.I.); 5 CRAS-MADRID Félix Rodríguez de la Fuente, Comunidad de Madrid, Ctra. Soto de Viñuelas s/n, Tres Cantos, 28760 Madrid, Spain; silvia.villaverde@ext.madrid.org

**Keywords:** *Chlamydia psittaci*, West Nile virus, wild birds, zoonosis, passive surveillance, One Health

## Abstract

Wild birds are relevant reservoirs and sentinels for zoonotic pathogens such as *Chlamydia psittaci* and West Nile virus (WNV), both of which can affect animal and public health. Wildlife rehabilitation centers (WRCs) offer unique opportunities for passive surveillance of emerging and re-emerging infectious diseases, particularly in urban and peri-urban settings. From 2013 to 2022, a total of 1024 bird samples were collected upon admission to WRCs in the Community of Madrid, Spain. Oropharyngeal and cloacal swabs, as well as tissue samples, were tested using real-time PCR targeting the ompA gene of *C. psittaci* and the 3’NC region of WNV. One sample tested positive for *C. psittaci* by real-time PCR in 2021, yielding a positivity rate of 0.22% (95% CI: 0.01–1.19). No positive cases were detected during the remaining years of the study. All samples tested negative for WNV over the nine-year period. The low detection rate suggests limited circulation of these pathogens among wild birds in central Spain, though it may partly reflect the variability inherent to passive surveillance and sample-type heterogeneity. However, continued surveillance is warranted, especially in high-risk avian species and personnel occupationally exposed in avian rehabilitation facilities using expanded sample sizes and complementary diagnostic tools. Extending monitoring beyond the typical vector season and increasing testing of sensitive tissues, particularly for WNV, may further enhance detection sensitivity and strengthen early-warning capacity. These efforts are essential to improve early detection and risk assessment within a One Health framework.

## 1. Introduction

Wild birds are recognized as key natural reservoirs for a wide range of zoonotic pathogens, including both bacterial and viral agents. Their ability to migrate over long distances and adapt to anthropogenically modified environments facilitates the transboundary spread of infectious agents, making them critical targets for wildlife disease surveillance under a One Health framework.

Among the zoonotic pathogens of avian origin, *Chlamydia psittaci* and West Nile virus (WNV) stand out due to their capacity to circulate silently in bird populations and occasionally spill over into humans and domestic animals. Despite differing in taxonomy and transmission mechanisms, both agents share ecological traits that enable their persistence and dissemination in wild bird communities.

*Chlamydia* is a genus of Gram-negative bacteria distributed globally, known for its obligatory intracellular nature, parasitizing eukaryotic cells. Birds are the primary hosts for multiple species of *Chlamydia*. Within this genus, there is variation in host specificity, with some species restricted to a single host, while others infect a wide range of wild and domestic animals, including humans. The most well-known species is *C. psittaci*, a zoonotic bacterium that affects a wide variety of birds, livestock, pets, and humans [1]. It is particularly prevalent in poultry, captive parrots, cockatoos, doves, and pigeons [2], which explains why it is mostly studied in humans, poultry, and pet birds [3]. Symptoms can range from asymptomatic to those involving ocular, respiratory, and gastrointestinal signs, with intermittent bacterial shedding, particularly during stressful situations (e.g., migration, breeding, illness) [4]. Zoonotic transmission occurs by the inhalation of respiratory secretion or dried feces dispersed in the air [4]. The severity of human chlamydiosis (also called psittacosis) varies depending on the virulence of the strain, with infections potentially resulting in severe respiratory disease [5]. In recent years, the Chlamydiaceae family has expanded with the identification of several new avian species, including *Chlamydia gallinacea*, *Chlamydia avium* and *Chlamydia buteonis*, which has challenged previous conceptions of host specificity and disease ecology [2,3,6]. These findings, alongside reports of novel transmission routes, such as the suspected spillover from wild birds to horses and humans, have renewed interest in the role of wild birds in chlamydial epidemiology [7]. Although its zoonotic potential is well recognized, *C. psittaci* remains largely underestimated by both healthcare professionals and public health authorities, particularly in Europe, where human cases often go undiagnosed due to nonspecific symptoms and limited awareness [5,7]. Systematic reviews indicate that its contribution to community-acquired pneumonia (CAP) is greater than generally assumed, with prevalence estimates in hospitalized patients ranging from around 1% to as high as 6.7% in some reports [8]. In Spain, sporadic outbreaks and familial clusters have been documented, particularly among individuals exposed to pet or wild birds [9,10]. However, no national surveillance program or seroprevalence studies are currently in place, suggesting that the burden of psittacosis is likely underestimated.

West Nile virus (WNV) is a re-emerging zoonotic, arthropod-borne virus of the Flaviviridae family, primarily transmitted by *Culex* spp. mosquitoes in an enzootic cycle involving birds as amplifying hosts. First identified in Uganda in 1937, WNV has expanded its geographic range significantly, and now circulates in multiple continents, including Europe, where prevalence has increased in the last decade. In Spain, WNV has been detected in birds, horses, and humans. The first human case was retrospectively diagnosed in 2004 [11], with outbreaks occurring from 2010 onward, particularly in Andalucía and Extremadura. The largest WNV epidemic reported in Spain occurred in 2020, with 77 confirmed human cases and at least eight deaths [12]. Human cases have been reported annually ever since, with a marked increase in both case numbers and fatalities in 2024 [13]. Similarly, equine cases have also been reported annually since their initial detection in 2010, including a seropositive horse in Madrid [14]. WNV has been recorded in over 392 bird species [15]. In Spain, the first indirect evidence of transmission in wild birds emerged in 2003 through a serological survey of the Common Coot (*Fulica atra*), a partially migratory aquatic species in Southern Spain [16]. Subsequent studies have confirmed WNV presence in both wild and captive birds using direct and indirect detection methods [17,18,19,20]. WNV comprises at least eight phylogenetic lineages, with lineages 1 (L1) and 2 (L2) being the most widespread and associated with major outbreaks. L2 has been increasingly detected in Central and Eastern Europe since 2004, where it is responsible for most human cases. In Spain, L1 has been circulating since decades in the south, west, and central regions [21], while L2 has been present in the northeast of the country for eight years and appears to be advancing southward along the Valencian coast [22].

As natural reservoirs for *C. psittaci* and WNV, wild birds are able to reintroduce these pathogens into domestic animal and human populations due to their wide-ranging movements and ability to forage in urban and peri-urban areas [23]. Consequently, monitoring and surveillance of zoonotic pathogens in wild birds have become essential for preventive measures, particularly in regions with previous evidence of pathogen circulation [24].

Wildlife Rehabilitation Centers (WRCs) represent a valuable yet underutilized tool for disease surveillance, as they admit birds from diverse environments, often presenting clinical signs or debilitated conditions that increase the likelihood of pathogen detection [25]. Consequently, WRCs can act as passive surveillance networks and provide early warnings for the circulation of zoonotic pathogens in wildlife.

Despite their potential, few studies have investigated *C. psittaci* in wild birds in Spain, with most focusing on urban pigeons, poultry, and occasionally waterfowl, thus leaving a considerable knowledge gap concerning other wild avian species [26,27,28]. This is particularly relevant considering that certain wild birds, such as scavengers and migratory species, may act as ecological bridges between remote natural ecosystems and human-dominated landscapes, thereby playing a key role in pathogen dispersal [29,30]. Additionally, monitoring invasive wild bird species is also critical, as their presence may facilitate the (re-)emergence of zoonotic diseases, as previously highlighted by Andersen et al. (2004) [31]. Moreover, few studies have assessed the risk of zoonotic transmission to WRC personnel, despite evidence of exposure scenarios. Similarly, although seropositive juvenile birds have recently been detected in central Spain, suggesting active local flavivirus [32], data on WNV circulation in the region remain scarce. Given the importance of this pathogen in the country, sustained surveillance efforts are essential to anticipate and mitigate potential outbreaks. Therefore, the aim of the present study was to determine the prevalence of *C. psittaci* and WNV in wild birds admitted to a Wildlife Rehabilitation Center in central Spain. By contributing novel data on this pathogen’s circulation in poorly studied avian hosts, this research seeks to enhance our understanding of the epidemiological role of wild birds in those zoonotic agents’ transmission, and to inform future wildlife and public health strategies.

## 2. Materials and Methods

### 2.1. Study Area and Bird Population

This study was part of a nine-year (2013–2022) wildlife surveillance program in Madrid, including samples obtained from the Soto de Viñuelas Wildlife Rehabilitation Center (WRC) to determine the role of wildlife in zoonotic pathogen transmission. The center is dedicated to the conservation and recovery of native fauna from Madrid regions that are injured or found ill, with the goal of returning them to their natural environment when possible. Ethical review and approval were not required for this study, as all procedures were conducted as part of routine diagnostic, clinical care, and rehabilitation activities at the authorized Wildlife Rehabilitation Center. No experimental procedures or interventions exceeding standard veterinary practice were performed.

A total of 1024 samples (520 from live animals and 504 post-mortem), were collected from different bird species, listed in Table 1 (see also Supplementary Tables S2 and S4). Of these, 464 (45.3%) were tested for *C. psittaci* and 560 (54.7%) for WNV.

A total of 49 avian species from 14 orders were examined (Table 1). The most represented groups for both *C. psittaci* and WNV were Ciconiiformes (23.3% and 22.3%, respectively), dominated by the White Stork (*Ciconia ciconia*); Accipitriformes (20.3% and 21.1%), mainly represented by the Black Kite (*Milvus migrans*); and Psittaciformes (18.8% and 19.6%), largely due to the Monk Parakeet (*Myiopsitta monachus*). Other orders with a moderate contribution included Charadriiformes (13.1% and 11.4%), Anseriformes (5.4% and 4.5%), and Strigiformes (5.4% and 6.2%). Falconiformes, Passeriformes, Suliformes, Pelecaniformes, and Gruiformes accounted for smaller proportions, while Apodiformes and Podicipediformes were only marginally represented (<1%). This distribution highlights that most samples originated from a few dominant avian orders, consistent across both pathogens.

### 2.2. Samples for Pathogen Detection

Samples collected from birds included cloacal swabs (195), tracheal swabs (205), cloacal and tracheal swab pools (502), and brain tissue (122). Sampling occurred throughout the year, and data on month, sex and age were recorded when available; because these variables were not systematically recorded for all individuals, the denominators used for percentage calculations are indicated in the corresponding tables. Swabs were collected per individual and stored in 1 mL of PBS at −80 °C, while brains from deceased birds were sent frozen and stored at −80 °C before processing at VISAVET Health Surveillance Centre for pathogen detection. The subset of individuals tested for each pathogen varied according to sample availability and diagnostic requirements. Testing was not based on clinical suspicion; instead, sampling followed the routine passive-surveillance workflow of the WRC. Selection was opportunistic but integrated into a systematic process, with samples collected from live and deceased birds depending on their condition and the diagnostic needs defined by the regional surveillance program at each period. Consequently, some individuals could not be tested for a given pathogen because the appropriate sample type (e.g., brain tissue for WNV) was unavailable, non-viable, or incompatible with the required diagnostic protocol, resulting in differing sample sizes across pathogens.

### 2.3. Molecular Screening

Total DNA from swabs was extracted using the QIAamp Minelute Virus Spin kit (Qiagen, Hilden, Germany), while DNA from brain tissues was obtained with the RNeasy Lipid Tissue kit (Qiagen, Hilden, Germany), following the manufacturer’s instructions. All procedures were performed under biosafety level 3 (BSL-3) conditions. Real time PCR targeting *C. psittaci* was performed on swabs DNA using the primers and probe described by Pantchev et al. (2009) [33] (Table 2) on a CFX96 thermal cycler (Bio-rad, Hercules, CA, USA). For every sample, a 25 μL reaction mix was prepared, including 5 µL of the sample, 5 μL of the master mix Quantifast Pathogen (Qiagen, Hilden, Germany), 0.75 µL of each primer (20 µM) and 0.5 µL of the probe (20 µM). Positive and negative PCR controls (*C. psittaci* in-house strain NED15/1918, West Nile Virus L1 NY99EqRNA and West Nile Virus L2 B956 RNA) and an internal control to detect inhibitions were added, with 1.5 μL of 10× Internal Control Assay and Internal control DNA both from Qiagen. The cycle conditions included 95 °C for 5 min, followed by 40 cycles of 95 °C for 15 s and 60 °C for 1 min.

For WNV detection, nucleic acids extracted from brain tissues were analyzed by RT-PCR following the protocol described by Jiménez-Clavero et al. (2006) [34], using the primers and probes listed in Table 2. The assay was performed using the commercial Quantifast Pathogen RT-PCR kit (Qiagen, Hilden, Germany). In brief, 2 µL of purified RNA was combined with 5 µL of 2× Quantifast RT-PCR Master Mix, 0.25 µL of Quantifast RT mix, 0.5 µL of WNV-specific primers at 20 µM concentration, and 0.25 µL of the probes at 20 µM. An internal control to detect inhibitions was added, with 1.5 μL of 10× Internal Control Assay and Internal control DNA, both from Qiagen (Qiagen, Hilden, Germany). RNase-free water was added to reach a total reaction volume of 25 µL. The thermal cycling protocol included an initial reverse transcription at 50 °C for 20 min, followed by a 5 min of polymerase activation step at 95 °C, and 45 amplification cycles of 95 °C for 15 s and 60 °C for 1 min. Reaction were run on a CFX96 thermal cycler (Bio-Rad, Hercules, CA, USA).

### 2.4. Statistical Analysis

Qualitative variables were described using frequencies and percentages. The proportion of positive cases was calculated as positives divided by the number tested (%), with 95% confidence intervals estimated using the exact binomial (Clopper–Pearson) method. Analyses were conducted in IBM SPSS Statistics (v28). Given the very low number of positive detections for both pathogens and the incomplete recording of covariates such as age and sex, no formal risk-factor or regression analyses were performed.

## 3. Results

A total of 464 birds were tested for the presence of *C. psittaci* and 560 for WNV over the study period. The types of samples collected, seasonal distribution, and demographic characteristics (sex and age) of the birds tested for each pathogen are summarized in Table 3 (see also Supplementary Tables S1 and S3). Sampling was most frequent in the first quarter of the year for both pathogens, and pooled cloacal and tracheal swabs represented the predominant sample type. Adult individuals constituted most of the birds tested, with slightly more males than females recorded in both datasets. These patterns illustrate the seasonal concentration of sampling and the predominance of specific sample types that are inherent to passive surveillance at WRCs, as well as the incomplete recording of some demographic variables.

Real-time PCR detected a single *C. psittaci* positive in 2021, yielding an overall positivity of 0.22% (95% CI: 0.01–1.19). No positives were identified in any other year of the study period (Table 4).

*C. psittaci* was detected in an adult white stork (*C. ciconia*) admitted in January 2021, which arrived together with 27 conspecifics. Considering the white stork population analyzed (n = 83), the intraspecific prevalence was 1/83 = 1.20% (95% CI: 0.03–6.53%).

No positive cases of WNV were detected among the 560 samples tested (0.00% overall; 95% exact upper limit ≈ 0.66%), indicating that all samples collected across 2013–2022 were negative for the virus.

## 4. Discussion

Key epidemiological factors linking wild birds to zoonotic diseases include their migratory movements and their capacity to adapt to urban and anthropogenic environments, thereby enhancing opportunities for contact with humans and domestic animals. Wild birds have long been proposed as natural reservoirs of *C. psittaci*, a zoonotic agent traditionally associated with captive psittacines. Although this association remains important, growing evidence indicates a broader host range that includes both wild and domestic bird species [35]. Nevertheless, direct evidence supporting the role of free-living wild birds as reservoirs is limited [5], and empirical data on their contribution to the epidemiology of *C. psittaci* remain scarce.

Recent advances in avian chlamydial research have identified several novel *Chlamydia* species, broadening the understanding of host range and pathogen diversity in wild birds [3]. Moreover, new potential transmission routes involving not only birds but also mammals such as horses and humans have been described, underscoring the complex epidemiological interactions and the necessity for comprehensive surveillance encompassing diverse wild avifauna and their environments.

The migratory behavior of many wild bird species facilitates the dissemination of pathogens across large geographic areas, while their adaptation to anthropogenic environments, such as urban parks and landfills, may increase contact rates with humans and domestic animals, elevating zoonotic risk [30].

The present study addresses a significant knowledge gap, as most previous research on avian chlamydiosis in Spain has focused on domestic birds, pigeons, or waterfowl, rather than on a broader spectrum of wild species. By leveraging the unique setting of a Wildlife Rehabilitation Center (WRC) in Madrid (Central Spain), this work contributes valuable epidemiological insight. As highlighted by Stitt et al. (2007) [25], WRCs are ideal platforms for pathogen surveillance due to the diversity of species admitted and their broad geographic origins. Birds admitted due to illness or injury may also be more likely to present active infections, thus improving pathogen detection sensitivity.

Most samples analyzed for both *C. psittaci* and WNV originated from birds of the orders Ciconiiformes, Accipitriformes, and Psittaciformes, underscoring their relevance in health surveillance and their potential role in the maintenance and transmission of these pathogens. Conversely, orders such as Apodiformes and Podicipediformes were scarcely represented, which could be due to a lower frequency of admission or capture in wildlife rehabilitation centers, although their epidemiological relevance should not be underestimated. This distribution emphasizes that surveillance efforts tend to concentrate on a limited number of avian groups, which may bias our understanding of pathogen circulation in the broader bird community.

*C. psittaci* was detected only in a white stork (*C. ciconia*) admitted to the WRC in 2021. This individual was found in Perales del Río, approximately 11 km from Madrid’s largest landfill (Valdemingómez), an area known to attract large flocks of scavenging birds. The white stork is a long-distance migratory species whose population in Spain has grown significantly in recent decades, partly due to the year-round availability of anthropogenic food sources, such as landfills [36,37,38,39]. This trend has resulted in an increasing number of storks admitted to rehabilitation centers [40], a pattern also observed in the current study, where *C. ciconia* was the most represented species (n = 83). Their high density in human-modified environments, opportunistic foraging (including the frequent use of landfills), and interactions with other species such as gulls that frequent similar habitats highlight the potential role of white storks as sentinel species in zoonotic pathogen surveillance [30]. Such ecological traits may facilitate interspecies transmission of pathogens, including *C. psittaci*. Nonetheless, in this study, the variation in sample sizes across years, particularly the limited number of birds analyzed between 2017 and 2019, restricts the evaluation of temporal trends. Within these limitations, the results suggest that *C. psittaci* was either absent or circulating at very low levels in this population during the study period. Overall prevalence of *C. psittaci* in the sampled birds was very low, contrasting with a global pooled prevalence of 19.5%, constant since 2012, estimated in a recent systematic review and meta-analysis [41]. In the present study, no positives were found among psittacines (n = 87) despite this order being consistently identified as a primary reservoir in other regions particularly in outbreaks linked to pet birds and aviaries [7,42]. Prevalence rates ranging from 2.5% to 10.3% have been reported in parrots across Costa Rica (3.4%), Poland, and Thailand [43,44,45]. Moreover, a recent survey detected *C. psittaci* in 23.8% of monk parakeets in Sevilla and Madrid [46]. Similarly, previous studies reported prevalence rates of 13–96% in Columbiformes [35], 55.2% in Anatidae and 11.8% in Corvidae [47], and lower rates in Accipitridae (1.3%) and Passerines (2.9%) [48]. In our dataset, the limited representation of these groups (e.g., only three Columbiformes sampled) and small sample sizes per species likely explain the absence of positives.

Comparisons with studies in Spain also illustrate wide variability: prevalence values ranged from 2.6% to 52.6% in pigeons [27,28], 13% in waterfowl [26], and 25% in gulls [29]. In contrast, large-scale surveillance from Switzerland and Australia, each involving more than 600 birds, reported prevalence rates below 1% [49,50]. Notably, Stalder and collaborators in 2020 [50] analyzed 316 birds, including five white storks, all of which tested negative. Methodological differences may explain some discrepancies, as fecal swabs are considered less sensitive than choanal swabs [51].

Other taxa also tested negative in our dataset. No raptors were positive, which may reflect the heterogeneity in reported prevalence among studies and the fact that, although raptors have been shown to harbour *Chlamydia* spp., they have historically received less targeted surveillance compared to psittacines and pigeons [3]. Expanding surveillance in these groups and across different geographic regions is warranted. In line with this, sporadic detection has been reported in scavenging species such as black vultures in Patagonia, where prevalence was 5.3% [52]. Prevalence may also vary with host age or health status: nestlings and juveniles often show lower infection rates [53], while severe acute disease could lead to rapid mortality, reducing the likelihood of detection in wild populations [54].

Finally, species strongly associated with anthropogenic environments, including white storks, gulls, and pigeons, merit particular attention, as they exploit landfills and urban habitats where opportunities for cross-species transmission are enhanced [29,30]. Despite this, few studies have specifically examined *Chlamydia* prevalence in white storks, with most research focusing on pigeons or captive birds.

Beyond ecological implications, these findings underscore the occupational risk for personnel in rehabilitation centers, where close contact with potentially infected birds is frequent. Cases of zoonotic transmission to workers have been documented [55,56], reinforcing the need for biosafety measures and sustained surveillance despite the overall low prevalence detected.

On the other hand, WNV RNA was not detected in the bird samples included in this study, although several limitations must be considered. WNV viraemia in birds is typically short-lived, lasting approximately 5–7 days post-infection [57], which further complicates the identification of acute infections when relying on samples collected outside this narrow temporal window. The type of sample used in this study (primarily cloacal and tracheal swabs) may have reduced detection sensitivity, as viral loads in these matrices are generally lower than in serum or tissue and can vary across avian species [58,59,60,61,62], which could lead to underestimation of true prevalence. Additionally, although brain and internal organs (e.g., heart, spleen, lungs) are considered more sensitive for WNV detection [58], their collection is more invasive and not always feasible in passive surveillance systems. Together, these factors could have contributed to an underestimation of the true prevalence of WNV in the studied population.

In Spain, there is a notable lack of published data on the circulation of WNV in wild birds in the central region. The recent report by Williams and collaborators (2024) [32], which documented the detection of flaviviruses in birds in the Madrid region, highlights the need for continued and enhanced surveillance. Considering that several avian species within the Orders Passeriformes, Strigiformes, and Columbiformes, commonly found in the study area, are recognized as amplifying hosts for WNV [63,64,65], ongoing monitoring remains crucial. Furthermore, surveillance systems should balance diagnostic sensitivity with feasibility and cost-effectiveness [66,67], potentially integrating both molecular and serological methods to better capture temporal and spatial patterns of virus circulation. Given these considerations, our findings underscore the importance of maintaining WNV surveillance in central Spain, while also refining sampling strategies to improve detection efficiency. Moreover, surveillance efforts should not be limited to the typical vector season, as recent evidence from Northern Italy has demonstrated off-season circulation of WNV and USUV in wild birds, supporting the possibility of flavivirus activity outside the typical vector season [68]. This finding reinforces the importance of sustained year-round surveillance and complements our observations in central Spain. The implementation of a surveillance program for zoonotic pathogens in wild birds admitted to WRCs has proven to be a valuable tool for monitoring avian reservoirs of infectious diseases with public health relevance. These centers provide a unique opportunity to monitor the circulation of infectious agents among wild avian populations, many of which may serve as reservoirs or sentinels for emerging diseases.

After a decade of monitoring in central Spain, our findings suggest that wild birds admitted to WRCs are not currently significant reservoirs of *C. psittaci* or WNV. However, given the dynamic nature of pathogen circulation, ongoing and expanded surveillance remains essential, especially in species with migratory or scavenging behaviors that may play a role in the transmission of zoonotic agents.

From a statistical perspective, the absence of WNV-positive birds among 560 samples implies a very low upper 95% confidence limit for the apparent prevalence in this surveillance setting. Under a simple binomial model with independent sampling and assuming perfect test sensitivity, the probability of detecting at least one positive bird would be very high if the true prevalence were around 1%, suggesting that sustained moderate prevalence in the tested population is unlikely

Although passive surveillance inherently leads to variability in the number of samples and sample types obtained across years, our dataset represents one of the most sustained and taxonomically diverse avian surveillance efforts conducted in central Spain. Nonetheless, some methodological factors should be considered when interpreting the very low detection rates observed, particularly the predominance of pooled swabs, the limited availability of brain tissues, and temporal fluctuations in sample availability, all of which may have reduced analytical sensitivity and hindered the detection of short-lived infections. Despite these limitations, the absence of WNV detection over a decade and the identification of only one *C. psittaci*-positive case provide valuable baseline information suggesting scarce or sporadic pathogen circulation in the wild bird species admitted to WRCs during this period, offering meaningful long-term insight in a region where data remain limited.

Moreover, although the number of brain samples in our study is more limited, the use of cloacal and tracheal swabs is a well-established and scientifically validated approach for WNV surveillance in birds. Several studies have demonstrated that oral, cloacal, and tracheal swabs can provide reliable detection of WNV, with sensitivities comparable to brain tissue, particularly when molecular methods such as RT-PCR are employed. Komar et al. in 2002 showed that cloacal and oral swabs collected post-mortem from corvids yielded high detection rates of WNV RNA, supporting their value as practical alternatives to brain tissue in large-scale monitoring programs [69]. Similarly, Stone and collaborators reported that RT-PCR on cloacal and oral swabs achieved sensitivity up to 91% in corvids, highlighting their utility for epidemiological studies [70]. Additionally, Nemeth et al. (2009) explored the use of nonvascular feathers as an additional non-invasive sample type, further reinforcing the feasibility of alternatives to brain tissue [71]. Therefore, while brain tissue remains the most sensitive matrix for WNV confirmation, the predominance of swab samples in our dataset reflects the routine and safe sampling of live birds entering the Center, balancing diagnostic accuracy with biosafety and logistical considerations.

To improve detection and epidemiological insight, future surveillance should prioritize increasing sample sizes, focusing on high-risk taxa (e.g., pigeons, parrots, gulls, raptors, scavengers), and employing multiple diagnostic approaches and sample types. These efforts will enhance sensitivity and strengthen early warning systems, contributing to integrated One Health strategies aimed at preventing zoonotic outbreaks and supporting both wildlife conservation and public health initiatives.

## 5. Conclusions

This study presents a decade-long (2013–2022) surveillance of *C. psittaci* and WNV in wild birds admitted to Wildlife Rehabilitation Centers in central Spain. Real-time PCR performed in 1024 bird samples detected one *C. psittaci* positive bird and no WNV-positive samples across the study period, indicating a very low apparent prevalence of *C. psittaci* and absence of detectable WNV circulation. While variability inherent to passive surveillance and sample-type heterogeneity may have influenced detection sensitivity, the large multispecies dataset collected over ten years provides valuable insight into pathogen dynamics in central Spain. The very low apparent circulation of *C. psittaci* and the absence of WNV detection in this long-term cohort suggest that these pathogens were not actively circulating at detectable levels in the birds admitted to WRCs during the study period. Nevertheless, continued surveillance using standardized sampling and expanded testing of sensitive tissues remains essential to better assess low-level or sporadic circulation. Future efforts should focus on sentinel taxa (e.g., storks, raptors, psittacines), incorporate complementary diagnostics such as serology, increase brain sampling for WNV, and extend monitoring beyond the typical vector season. Wildlife Rehabilitation Centers thus continue to represent valuable platforms for early detection and risk assessment of zoonotic pathogens.

## Figures and Tables

**Table 1:** Bird orders, common names, and number of individuals analyzed for *C. psittaci* and WNV detection in the present study.

Order of Birds	Common Bird Name (No. Tested for WNV and *C. psittacii*)	*C. psittaci* n (%)	WNV n (%)
Ciconiiformes	White Stork (183), Cattle Egret (24), Little Egret (24), Little Bittern (2)	108 (23.3%)	125 (22.3%)
Accipitriformes	Black Kite (56), Common Buzzard (44), Griffon Vulture (30), Red Kite (25), Booted Eagle (22), Spanish Imperial Eagle (10), Black Vulture (10), Golden Eagle (5), Western Marsh Harrier (4), Eurasian Sparrowhawk (4), Northern Goshawk (2)	94 (20.3%)	118 (21.1%)
Psittaciformes	Monk Parakeet (181), Cockatiel (4), Peach-faced Lovebird (4), Blue-fronted Amazon (2), Lovebird (2), Burrowing Parrot (2), Grey Parrot (2),	87 (18.8%)	110 (19.6%)
Charadriiformes	Mediterranean Gull (51), Lesser Black-backed Gull (53), Black-headed Gull (19), Common Snipe (2)	61 (13.1%)	64 (11.4%)
Anseriformes	Mallard (38), Egyptian Goose (8), Domestic Goose (2), Mute Swan (2)	25 (5.4%)	25 (4.5%)
Strigiformes	Eurasian Eagle-Owl (55), Little Owl (5)	25 (5.4%)	35 (6.2%)
Falconiformes	Lesser Kestrel (27), Peregrine Falcon (10), Common Kestrel (5)	17 (3.7%)	25 (4.5%)
Passeriformes	House Sparrow (8), Eurasian Magpie (13), Song Thrush (4), European Robin (4), Common Raven (2), Great Grey Shrike (2)	14 (3.0%)	19 (3.4%)
Suliformes	Great Cormorant (24)	11 (2.4%)	13 (2.3%)
Pelecaniformes	Grey Heron (12), Common Spoonbill (4), Black-crowned Night Heron (2)	9 (1.9%)	9 (1.6%)
Gruiformes	Great Bustard (15), Common Crane (2), Common Coot (2)	8 (1.7%)	11 (2.0%)
Columbiformes	Rock Pigeon (5), Wood Pigeon (2)	3 (0.6%)	4 (0.7%)
Apodiformes	Common Swift (2)	1 (0.2%)	1 (0.2%)
Podicipediformes	Great Crested Grebe (2)	1 (0.2%)	1 (0.2%)
TOTAL		464 (100%)	560 (100%)

**Table 2:** Primer sequences and references for *C. psittaci* and WNV detection.

Pathogen and Primer	Nucleotide Sequence (5′ to 3′) and Labeling	Reference
*Chlamydia psittaci*		
CppsOMP1-F	CACTATGTGGGAAGGTGCTTCA	
CppsOMP1-R	CTGCGCGGATGCTAATGG	Pantchev et al., 2009 [33]
CppsOMP1-S	FAM ^a^-CGCTACTTGGTGTGACBHQ1 ^b^	
West Nile Virus		
WN-LCV-F1	GTGATCCATGTAAGCCCTCAGAA	
WN-LCV-R1	GTCTGACATTGGGCTTTGAAGTTA	Jiménez-Clavero et al., 2006 [34]
WN-LCV-S1	FAM ^a^-AGGACCCCACATGTT-MGB ^c^	
WN-LCV-S2	FAM ^a^-AGGACCCCACGTGCT-MGB ^c^	

^a^ FAM 6-carboxy-fluorescein. ^b^ Black hole quencher. ^c^ MGB (minor groove binding) probe.

**Table 3:** Summary of sample types, seasonal distribution, and demographic characteristics of birds tested for *C. psittaci* and WNV over a nine-year period.

Variable	*C. psittaci* (n = 464)	WNV (n = 560)
**Sample type**		
Pooled cloacal/tracheal	251 (54.1%)	251 (44.8%)
Tracheal swab	96 (20.7%)	109 (19.5%)
Cloacal swab	91 (19.6%)	104 (18.6%)
Brain	26 (5.6%)	96 (17.1%)
**Sampling by month**		
January	111 (23.9%)	118 (21.1%)
February	53 (11.4%)	74 (13.2%)
March	46 (9.9%)	64 (11.4%)
April	0 (0.0%)	51 (9.1%)
May	14 (3.0%)	21 (3.8%)
June	34 (7.3%)	36 (6.4%)
July	34 (7.3%)	44 (7.9%)
August	16 (3.4%)	25 (4.5%)
September	22 (4.7%)	28 (5.0%)
October	49 (10.6%)	47 (8.4%)
November	19 (4.1%)	31 (5.5%)
December	29 (6.3%)	21 (3.8%)
**Sex** *		
Female	54 (46.2%)	78 (45.6%)
Male	63 (53.8%)	93 (54.4%)
**Age** *		
Adults	186 (83.4%)	239 (80.5%)
Young	27 (12.1%)	48 (16.2%)
Juveniles	10 (4.5%)	10 (3.4%)

* Percentages for sex and age are calculated over the subset with available data.

**Table 4:** Total birds analyzed for *C. psitacii* and WNV per year and annual prevalences for the study period (2013–2022).

Year	*C. psittaci* Tested	*C. psittaci* % Positive (95% CI)	WNV Tested	WNV % Positive (95% CI)
2013	88	0.00% (0.00–4.11)	130	0.00% (0.00–2.80)
2014	38	0.00% (0.00–9.25)	55	0.00% (0.00–6.49)
2015	78	0.00% (0.00–4.62)	115	0.00% (0.00–3.16)
2016	40	0.00% (0.00–8.81)	40	0.00% (0.00–8.81)
2017	16	0.00% (0.00–20.59)	16	0.00% (0.00–20.59)
2018	2	0.00% (0.00–84.19)	2	0.00% (0.00–84.19)
2019	7	0.00% (0.00–40.96)	7	0.00% (0.00–40.96)
2020	61	0.00% (0.00–5.87)	61	0.00% (0.00–5.87)
2021	73	1.37% (0.03–7.40)	73	0.00% (0.00–4.93)
2022	61	0.00% (0.00–5.87)	61	0.00% (0.00–5.87)
TOTAL	464	0.22% (0.01–1.19)	560	0.00% (0.00–0.66)

## Data Availability

The original contributions presented in this study are included in the article/Supplementary Material. Further inquiries can be directed to the corresponding author.

## Supplementary Materials

The following supporting information can be downloaded at: https://www.mdpi.com/article/10.3390/microorganisms14010048/s1, Table S1: Distribution of samples tested for *Chlamydia* spp. by type, year, month, age, and sex (n = 464). Table S2: Distribution of bird species tested for *Chlamydia* spp. by order, number of samples, and percentage (n = 464). Table S3: Distribution of samples tested for WNV by type, year, month, age, and sex (n = 560). Table S4: Distribution of bird species tested for WNV by order, number of samples, and percentage (n = 560).

## Abbreviations

The following abbreviations are used in this manuscript:

WRC	Wildlife Rehabilitation Center
WNV	West Nile Virus

## References

[1] Hogerwerf L., Roof I., de Jong M.J.K., Dijkstra F., van der Hoek W. (2020). Animal sources for zoonotic transmission of psittacosis: A systematic review. BMC Infect. Dis..

[2] Sachse K., Laroucau K., Vanrompay D. (2015). Avian Chlamydiosis. Curr. Clin. Microbiol. Rep..

[3] Stokes H.S., Berg M.L., Bennett A.T.D. (2021). A Review of Chlamydial Infections in Wild Birds. Pathogens.

[4] Aaziz R., Laroucau K., Gobbo F., Salvatore D., Schnee C., Terregino C., Lupini C., Di Francesco A. (2022). Occurrence of Chlamydiae in Corvids in Northeast Italy. Animals.

[5] Beeckman D.S., Vanrompay D.C. (2009). Zoonotic Chlamydophila psittaci infections from a clinical perspective. Clin. Microbiol. Infect..

[6] Zocevic A., Vorimore F., Marhold C., Horvatek D., Wang D., Slavec B., Prentza Z., Stavianis G., Prukner-Radovcic E., Dovc A. (2012). Molecular characterization of atypical Chlamydia and evidence of their dissemination in different European and Asian chicken flocks by specific real-time PCR. Environ. Microbiol..

[7] Ravichandran K., Anbazhagan S., Karthik K., Angappan M., Dhayananth B. (2021). A comprehensive review on avian chlamydiosis: A neglected zoonotic disease. Trop. Anim. Health Prod..

[8] Hogerwerf L., De Gier B., Baan B., van der Hoek W. (2017). *Chlamydia psittaci* (psittacosis) as a cause of community-acquired pneumonia: A systematic review and meta-analysis. Epidemiol. Infect..

[9] Arenas-Valls N., Chacon S., Perez A., Del Pozo R. (2017). Atypical *Chlamydia psittaci* Pneumonia. Four Related Cases. Arch. Bronconeumol..

[10] Fernández P., Iborra M.A., Simón M., Segovia M. (2020). Brote de neumonía por *Chlamydia psittaci* en la Región de Murcia. Enfermedades Infecc. Microbiol. Clín..

[11] Kaptoul D., Viladrich P.F., Domingo C., Niubo J., Martinez-Yelamos S., De Ory F., Tenorio A. (2007). West Nile virus in Spain: Report of the first diagnosed case (in Spain) in a human with aseptic meningitis. Scand. J. Infect. Dis..

[12] European Center for Disease Prevention and Contro–ECDC (2020). Epidemiological Update: West Nile Virus Transmission Season in Europe. https://www.ecdc.europa.eu/en/news-events/epidemiological-update-west-nile-virus-transmission-season-europe-2020.

[13] European Center for Disease Prevention and Control—ECDC (2024). Communicable Disease Threats Report. https://www.ecdc.europa.eu/sites/default/files/documents/Communicable-disease-threats-report-week-50-2024.pdf.

[14] Abad-Cobo A., Llorente F., Barbero M.D.C., Cruz-Lopez F., Fores P., Jimenez-Clavero M.A. (2017). Serosurvey Reveals Exposure to West Nile Virus in Asymptomatic Horse Populations in Central Spain Prior to Recent Disease Foci. Transbound. Emerg. Dis..

[15] Williams R.A.J., Sanchez-Llatas C.J., Domenech A., Madrid R., Fandino S., Cea-Callejo P., Gomez-Lucia E., Benitez L. (2023). Emerging and Novel Viruses in Passerine Birds. Microorganisms.

[16] Figuerola J., Soriguer R., Rojo G., Gomez Tejedor C., Jimenez-Clavero M.A. (2007). Seroconversion in wild birds and local circulation of West Nile virus, Spain. Emerg. Infect. Dis..

[17] Jimenez-Clavero M.A., Sotelo E., Fernandez-Pinero J., Llorente F., Blanco J.M., Rodriguez-Ramos J., Perez-Ramirez E., Hofle U. (2008). West Nile virus in golden eagles, Spain, 2007. Emerg. Infect. Dis..

[18] Llorente F., Perez-Ramirez E., Fernandez-Pinero J., Soriguer R., Figuerola J., Jimenez-Clavero M.A. (2013). Flaviviruses in game birds, southern Spain, 2011–2012. Emerg. Infect. Dis..

[19] Lopez G., Jimenez-Clavero M.A., Vazquez A., Soriguer R., Gomez-Tejedor C., Tenorio A., Figuerola J. (2011). Incidence of West Nile virus in birds arriving in wildlife rehabilitation centers in southern Spain. Vector Borne Zoonotic Dis..

[20] Marzal A., Ferraguti M., Muriel J., Magallanes S., Ortiz J.A., Garcia-Longoria L., Bravo-Barriga D., Guerrero-Carvajal F., Aguilera-Sepulveda P., Llorente F. (2022). Circulation of zoonotic flaviviruses in wild passerine birds in Western Spain. Vet. Microbiol..

[21] Garcia San Miguel Rodriguez-Alarcon L., Fernandez-Martinez B., Sierra Moros M.J., Vazquez A., Julian Paches P., Garcia Villacieros E., Gomez Martin M.B., Figuerola Borras J., Lorusso N., Ramos Aceitero J.M. (2021). Unprecedented increase of West Nile virus neuroinvasive disease, Spain, summer 2020. Eurosurveillance.

[22] Aguilera-Sepulveda P., Napp S., Llorente F., Solano-Manrique C., Molina-Lopez R., Obon E., Sole A., Jimenez-Clavero M.A., Fernandez-Pinero J., Busquets N. (2022). West Nile Virus Lineage 2 Spreads Westwards in Europe and Overwinters in North-Eastern Spain (2017–2020). Viruses.

[23] Levison M.E. (2015). Diseases Transmitted by Birds. Microbiol. Spectr..

[24] Stallknecht D.E. (2007). Impediments to wildlife disease surveillance, research, and diagnostics. Curr. Top. Microbiol. Immunol..

[25] Stitt T., Mountifield J., Stephen C. (2007). Opportunities and obstacles to collecting wildlife disease data for public health purposes: Results of a pilot study on Vancouver Island, British Columbia. Can. Vet. J..

[26] Astorga R.J., Cubero M.J., Leon L., Maldonado A., Arenas A., Tarradas M.C., Perea A. (1994). Serological survey of infections in waterfowl in the Guadalquivir marshes (Spain). Avian Dis..

[27] Perez-Sancho M., Garcia-Seco T., Porrero C., Garcia N., Gomez-Barrero S., Camara J.M., Dominguez L., Alvarez J. (2020). A ten-year-surveillance program of zoonotic pathogens in feral pigeons in the City of Madrid (2005–2014): The importance of a systematic pest control. Res. Vet. Sci..

[28] Vazquez B., Esperon F., Neves E., Lopez J., Ballesteros C., Munoz M.J. (2010). Screening for several potential pathogens in feral pigeons (*Columba livia*) in Madrid. Acta Vet. Scand..

[29] Navarro J., Gremillet D., Afan I., Miranda F., Bouten W., Forero M.G., Figuerola J. (2019). Pathogen transmission risk by opportunistic gulls moving across human landscapes. Sci. Rep..

[30] Plaza P.I., Lambertucci S.A. (2017). How are garbage dumps impacting vertebrate demography, health, and conservation?. Glob. Ecol. Conserv..

[31] Andersen M.C., Adams H., Hope B., Powell M. (2004). Risk assessment for invasive species. Risk Anal..

[32] Williams R.A.J., Criollo Valencia H.A., Lopez Marquez I., Gonzalez Gonzalez F., Llorente F., Jimenez-Clavero M.A., Busquets N., Mateo Barrientos M., Ortiz-Diez G., Ayllon Santiago T. (2024). West Nile Virus Seroprevalence in Wild Birds and Equines in Madrid Province, Spain. Vet. Sci..

[33] Pantchev A., Sting R., Bauerfeind R., Tyczka J., Sachse K. (2009). New real-time PCR tests for species-specific detection of *Chlamydophila psittaci* and *Chlamydophila abortus* from tissue samples. Vet. J..

[34] Jiménez-Clavero M.A., Agüero M., Rojo G., Gómez-Tejedor C. (2006). A new fluorogenic real-time RT-PCR assay for detection of lineage 1 and lineage 2 West Nile viruses. J. Vet. Diagn. Investig..

[35] Harkinezhad T., Geens T., Vanrompay D. (2009). *Chlamydophila psittaci* infections in birds: A review with emphasis on zoonotic consequences. Vet. Microbiol..

[36] Aguirre J.I., Vergara P. (2009). Census methods for White stork (*Ciconia ciconia*): Bias in sampling effort related to the frequency and date of nest visits. J. Ornithol..

[37] Blanco G. (1996). Population dynamics and communal roosting of White Storks foraging at a Spanish refuse dump. Colon. Waterbirds.

[38] Schulz H. Der Welbestand des Weißstorch im Aufwind?-White Storks on the up?. Proceedings of the International Symposium on the White Stork.

[39] Molina B., Del Moral J.C. (2005). La Cigüeña Blanca en España.VI Censo Internacional (2004).

[40] Camacho M., Hernandez J.M., Lima-Barbero J.F., Hofle U. (2016). Use of wildlife rehabilitation centres in pathogen surveillance: A case study in white storks (*Ciconia ciconia*). Prev. Vet. Med..

[41] Sukon P., Nam N.H., Kittipreeya P., Sara-In A., Wawilai P., Inchuai R., Weerakhun S. (2021). Global prevalence of chlamydial infections in birds: A systematic review and meta-analysis. Prev. Vet. Med..

[42] Riccio M.B., García J.P., Chiapparrone M.L., Cantón J., Cacciato C., Origlia J.A., Cadario M.E., Diab S.S., Uzal F.A. (2024). Outbreak of Infection in a Commercial Psittacine Breeding Aviary in Argentina. Animals.

[43] Piasecki T., Chrzastek K., Wieliczko A. (2012). Detection and identification of *Chlamydophila psittaci* in asymptomatic parrots in Poland. BMC Vet. Res..

[44] Sheleby-Elias J., Solorzano-Morales A., Romero-Zuniga J.J., Dolz G. (2013). Molecular Detection and Genotyping of *Chlamydia psittaci* in Captive Psittacines from Costa Rica. Vet. Med. Int..

[45] Tripinichgul S., Weerakhun S., Kanistanon K. (2023). Prevalence and Risk Factors of Avian Chlamydiosis Detected by Polymerase Chain Reaction in Psittacine Birds in Thailand. J. Avian Med. Surg..

[46] López J., Mogedas M., Ballesteros C., Martín-Maldonado B., Sacristán I., García R., Ortiz J.C., Esperón F. (2023). Infectious agents present in monk parakeet (*Myiopsitta monachus*) and rose-ringed parakeet (*Psittacula krameri*) invasive species in the parks of Madrid and Seville, Spain. Front. Vet. Sci..

[47] Szymańska-Czerwińska M., Mitura A., Niemczuk K., Zaręba K., Jodełko A., Pluta A., Scharf S., Vitek B., Aaziz R., Vorimore F. (2017). Dissemination and genetic diversity of chlamydial agents in Polish wildfowl: Isolation and molecular characterisation of avian *Chlamydia abortus* strains. PLoS ONE.

[48] Blomqvist M., Christerson L., Waldenström J., Lindberg P., Helander B., Gunnarsson G., Herrmann B., Olsen B. (2012). *Chlamydia psittaci* in birds of prey, Sweden. Infect. Ecol. Epidemiol..

[49] Amery-Gale J., Legione A.R., Marenda M.S., Owens J., Eden P.A., Konsak-Ilievski B.M., Whiteley P.L., Dobson E.C., Browne E.A., Slocombe R.F. (2020). Surveillance for *Chlamydia* spp. with Multilocus Sequence Typing Analysis in Wild and Captive Birds in Victoria, Australia. J. Wildl. Dis..

[50] Stalder S., Marti H., Borel N., Mattmann P., Vogler B., Wolfrum N., Albini S. (2020). Detection of *Chlamydiaceae* in Swiss wild birds sampled at a bird rehabilitation centre. Vet. Rec. Open.

[51] Andersen A.A. (1996). Comparison of pharyngeal, fecal, and cloacal samples for the isolation of *Chlamydia psittaci* from experimentally infected cockatiels and turkeys. J. Vet. Diagn. Investig..

[52] Plaza P.I., Blanco G., Madariaga M.J., Boeri E., Teijeiro M.L., Bianco G., Lambertucci S.A. (2019). Scavenger birds exploiting rubbish dumps: Pathogens at the gates. Transbound. Emerg. Dis..

[53] Konicek C., Vodrážka P., Barták P., Knotek Z., Hess C., Račka K., Hess M., Troxler S. (2016). Detection of Zoonotic Pathogens in Wild Birds in the Cross-Border Region Austria-Czech Republic. J. Wildl. Dis..

[54] Tiyawattanaroj W., Lindenwald R., Mohr L., Günther E., Legler M. (2021). Monitoring of the infectious agent *Chlamydia psittaci* in common swifts (*Apus apus*) in the area of Hannover, Lower Saxony, Germany. Berl. Münch. Tierärztl. Wschr.

[55] Favier P., Wiemeyer G.M., Arias M.B., Lara C.S., Vilar G., Crivelli A.J., Ludvik H.K., Ardiles M.I., Teijeiro M.L., Madariaga M.J. (2024). *Chlamydia psittaci* Screening of Animal Workers from Argentina Exposed to Carrier Birds. Ecohealth.

[56] Ossa-Giraldo A.C., Usuga-Perilla X., Correa J.S., Segura J.A. (2023). *Chlamydia psittaci* seropositivity in workers exposed to birds and review of the literature: Evidence of circulation in Antioquia. Biomedica.

[57] Komar N., Langevin S., Hinten S., Nemeth N., Edwards E., Hettler D., Davis B., Bowen R., Bunning M. (2003). Experimental infection of North American birds with the New York 1999 strain of West Nile virus. Emerg. Infect. Dis..

[58] Atama N.C., Chestakova I.V., de Bruin E., van den Berg T.J., Munger E., Reusken C., Oude Munnink B.B., van der Jeugd H., van den Brand J.M.A., Koopmans M.P.G. (2022). Evaluation of the use of alternative sample types for mosquito-borne flavivirus surveillance: Using Usutu virus as a model. One Health.

[59] Langevin S.A., Bunning M., Davis B., Komar N. (2001). Experimental infection of chickens as candidate sentinels for West Nile virus. Emerg. Infect. Dis..

[60] Senne D.A., Pedersen J.C., Hutto D.L., Taylor W.D., Schmitt B.J., Panigrahy B. (2000). Pathogenicity of West Nile virus in chickens. Avian Dis..

[61] Swayne D.E., Beck J.R., Smith C.S., Shieh W.J., Zaki S.R. (2001). Fatal encephalitis and myocarditis in young domestic geese (Anser anser domesticus) caused by West Nile virus. Emerg. Infect. Dis..

[62] Swayne D.E., Beck J.R., Zaki S. (2000). Pathogenicity of West Nile virus for turkeys. Avian Dis..

[63] Brown C., O’Brien V. (2011). Are Wild Birds Important in the Transport of Arthropod-borne Viruses?. Ornithol. Monogr..

[64] Engel D., Jost H., Wink M., Borstler J., Bosch S., Garigliany M.M., Jost A., Czajka C., Luhken R., Ziegler U. (2016). Reconstruction of the Evolutionary History and Dispersal of Usutu Virus, a Neglected Emerging Arbovirus in Europe and Africa. mBio.

[65] Roiz D., Vazquez A., Ruiz S., Tenorio A., Soriguer R., Figuerola J. (2019). Evidence that Passerine Birds Act as Amplifying Hosts for Usutu Virus Circulation. Ecohealth.

[66] Artois M., Bengis R., Delahay R.J., Duchêne M.J., Duff J.P., Ferroglio E., Gortazar C., Hutchings M.R., Kock R.A., Leighton F.A. (2009). Wildlife Disease Surveillance and Monitoring. Management of Disease in Wild Mammals.

[67] Drewe J.A., Hoinville L.J., Cook A.J., Floyd T., Stark K.D. (2012). Evaluation of animal and public health surveillance systems: A systematic review. Epidemiol. Infect..

[68] Musto C., Tamba M., Calzolari M., Rossi A., Grisendi A., Marzani K., Bonilauri P., Delogu M. (2023). Detection of West Nile and Usutu Virus RNA in Autumn Season in Wild Avian Hosts in Northern Italy. Viruses.

[69] Komar N., Lanciotti R., Bowen R., Langevin S., Bunning M. (2002). Detection of West Nile virus in oral and cloacal swabs collected from bird carcasses. Emerg. Infect. Dis..

[70] Stone W.B., Therrien J.E., Benson R., Kramer L., Kauffman E.B., Eldson M., Campbell S. (2005). Assays to detect West Nile virus in dead birds. Emerg. Infect. Dis..

[71] Nemeth N., Young G., Burkhalter K., Brault A., Reisen W., Komar N. (2009). West Nile Virus Detection in Nonvascular Feathers from Avian Carcasses. J. Vet. Diagn. Investig..

